# Recurrent pneumothorax and intrapulmonary cavitary lesions in a male patient with vascular Ehlers-Danlos syndrome and a novel missense mutation in the COL3A1 gene: a case report

**DOI:** 10.1186/s12890-020-1164-4

**Published:** 2020-05-29

**Authors:** Tingting Wan, Jinyan Ye, Peiliang Wu, Mengshi Cheng, Baihong Jiang, Hailong Wang, Jianmin Li, Jun Ma, Liangxing Wang, Xiaoying Huang

**Affiliations:** 1 Division of Pulmonary Medicine, The First Affiliated Hospital of Wenzhou Medical University, Key Laboratory of Heart and Lung, Wenzhou, Zhejiang 325000 People’s Republic of China; 2 Division of Pathology, The First Affiliated Hospital of Wenzhou Medical University, Wenzhou, Zhejiang 325000 People’s Republic of China

**Keywords:** Vascular Ehlers-Danlos syndrome, Pneumothorax, Intrapulmonary lesions, COL3A1 gene, Collagen type III

## Abstract

**Background:**

Vascular Ehlers-Danlos syndrome (vEDS) is a rare autosomal dominant hereditary collagen disease caused by a defect or deficiency in the pro-α1 chain of type III procollagen encoded by the COL3A1 gene. Patients with vEDS rarely present with multiple pneumothoraces. The clinical features of this disease are not familiar to clinicians and are easily missed. We report a patient with a novel missense mutation in the COL3A1 gene (NM_000090.3: c.2977G > A) and hope to provide clinicians with valuable information.

**Case presentation:**

We reported the case of a young man presenting with frequent episodes of pneumothorax and intrapulmonary cavities and nodular lesions without arterial or visceral complications. His skin was thin and transparent, and the joints were slightly hypermobile. Whole-exome sequencing (chip capture high-throughput sequencing) revealed a heterozygous missense mutation in exon 41 of the COL3A1 gene (NM_000090.3: c.2977G > A), confirming the diagnosis of vEDS. vEDS remains a very rare and difficult diagnosis to determine.

**Conclusion:**

When a patient presents with recurrent pneumothorax, intrapulmonary cavities and nodular lesions, thin and transparent skin, and hypermobile joints, clinicians should consider the diagnosis of vEDS.

## Background

Vascular Ehlers-Danlos syndrome (vEDS, also known as type IV EDS) is characterized by thin, translucent skin, easy bruising, and a high risk of rupture of the arteries, bowel, and pregnant uterus [1]. The incidence of vEDS is approximately 1 in 150,000 [2]. In addition, it is often caused by mutations in the COL3A1 gene, which encodes the pro-α1 (III) chain of type III procollagen. Additionally, 90% of patients with vEDS present with external thoracic arterial dissection or rupture [1]. Patients with vEDS rarely present with multiple pneumothoraces.

In this report, we present a rare case of vEDS that manifested as recurrent pneumothorax and pulmonary lesions.

## Case presentation

A 24-year-old Chinese man presented to the hospital with a 6-day history of haemoptysis, cough and dizziness. He had a history of intermittent cough and blood-tinged sputum for 2 years but denied a history of infectious diseases, occupational exposures or foreign travel. He had no history of smoking. A history of penicillin allergy was noted. No significant medical or drug history was recorded.

Physical examination revealed a temperature of 37.2 °C, a heart rate of 143 beats/min, a blood pressure of 132/64 mmHg, a respiratory rate of 18 breaths/min, and a pulse oximetry value of 98% in ambient air. The patient was tall and slender with a height of 175 cm and a weight of 65 kg (body mass index of 21.22 kg/m^2^). Chest auscultation revealed decreased lung sounds on the right hemithorax. The clinical examination was otherwise normal.

Laboratory tests showed a white blood cell count of 9.45 × 10^9/L (neutrophils 67%, lymphocytes 23%, and eosinophils 1%), a haemoglobin level of 153 g/L and a platelet count of 231 × 10^9/L. The serum creatinine level, liver function tests, erythrocyte sedimentation rate and C-reactive protein level were all within normal limits. Tests for connective tissue disease with auto-antibodies, including antinuclear, anti-neutrophil cytoplasmic, and anti-glomerular basement membrane antibodies, were negative. The test for human immunodeficiency virus was negative.

**Fig. 1:**
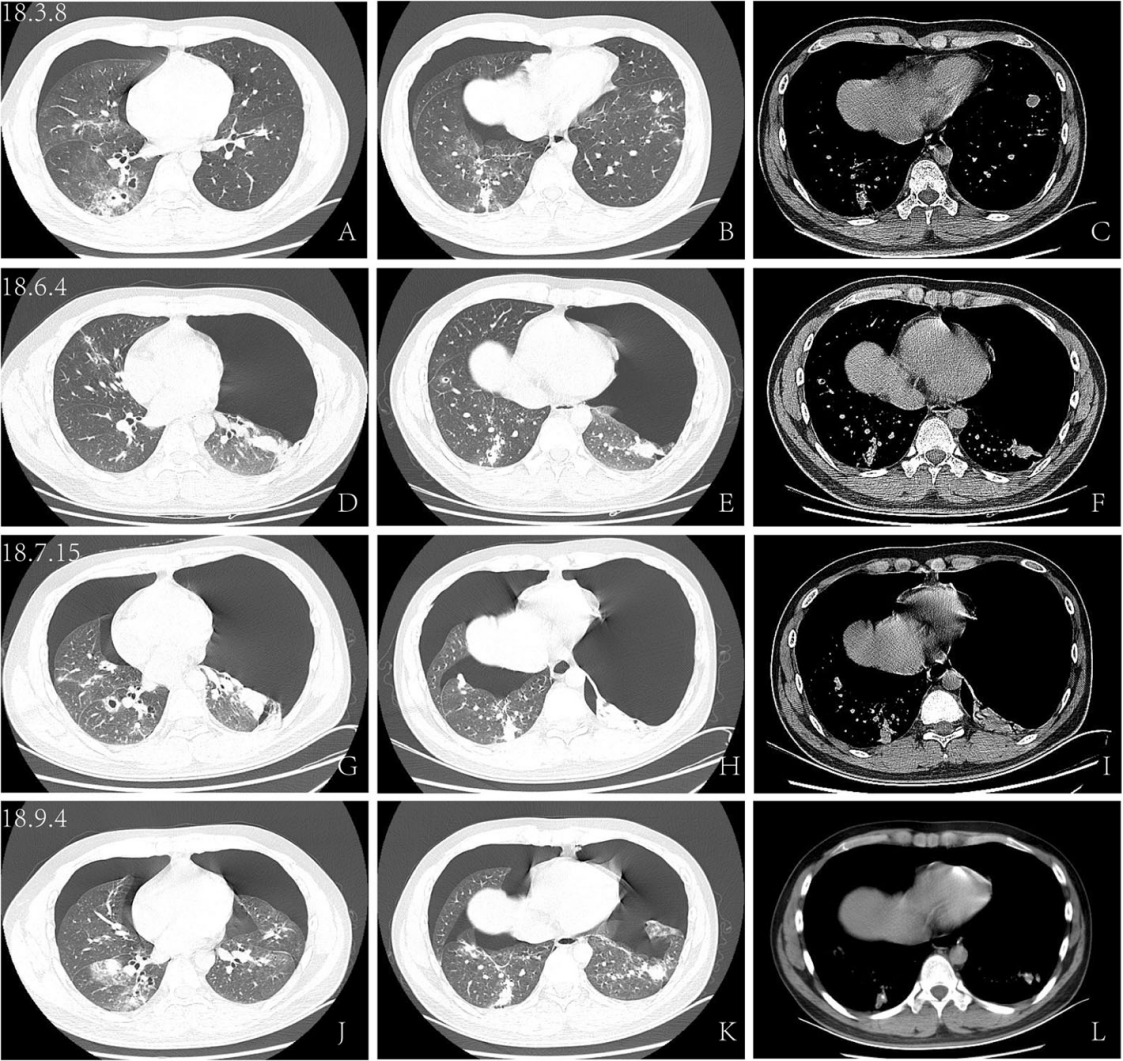
Computed tomography images of the chest. **a**, **b**, and **c** A CT scan of the lungs showed right-sided pneumothorax, small cavitary lesions in the right lower lobe and nodules in the left lung. **d**, **e** and **f** Chest CT images showing a large amount of gas in the left thoracic cavity and the left lung tissue compressed by approximately 60%. Both lungs displayed scattered, patchy and nodular high-density shadows, and some exhibited a ground-glass density. **g**, **h**, and **i** Chest CT images showing bilateral pneumothorax with the mediastinum deviated to the right, patch-like high-density shadows scattered in both lungs, and an ambiguous boundary. **j**, **k**, and **l** A CT scan of lungs showed gas on both sides of the chest cavity, and both lungs presented scattered nodules and patchy high-density shadows with unclear boundaries

A subsequent computed tomography (CT) scan revealed a right-sided pneumothorax, several small cavitary lesions in the right lower lobe and nodules in the left lung (Fig. 1a, b and c). An intercostal chest drain was inserted in the patient, with complete resolution of the pneumothorax. Bronchoscopy showed bronchial inflammatory disease. Due to a history of penicillin allergy, the patient refused further bronchial artery computed tomography angiography (CTA) and an enhanced chest CT examination. On day 12 of the hospital stay, he was discharged after the removal of the chest tube.

However, after 3 months, he visited our clinic again and complained of dyspnoea with a duration of 2 days. A high-resolution computed tomography (HRCT) scan of the chest was performed, which showed a large amount of gas in the left thoracic cavity, and the left lung tissue was compressed by approximately 60%. Both lungs had scattered patchy and nodular high-density shadows, and some shadows exhibited ground-glass density (Fig. 1d, e and f). The left-sided pneumothorax was managed with an intercostal chest drain. Both occurrences were diagnosed as primary spontaneous pneumothorax.

**Fig. 2:**
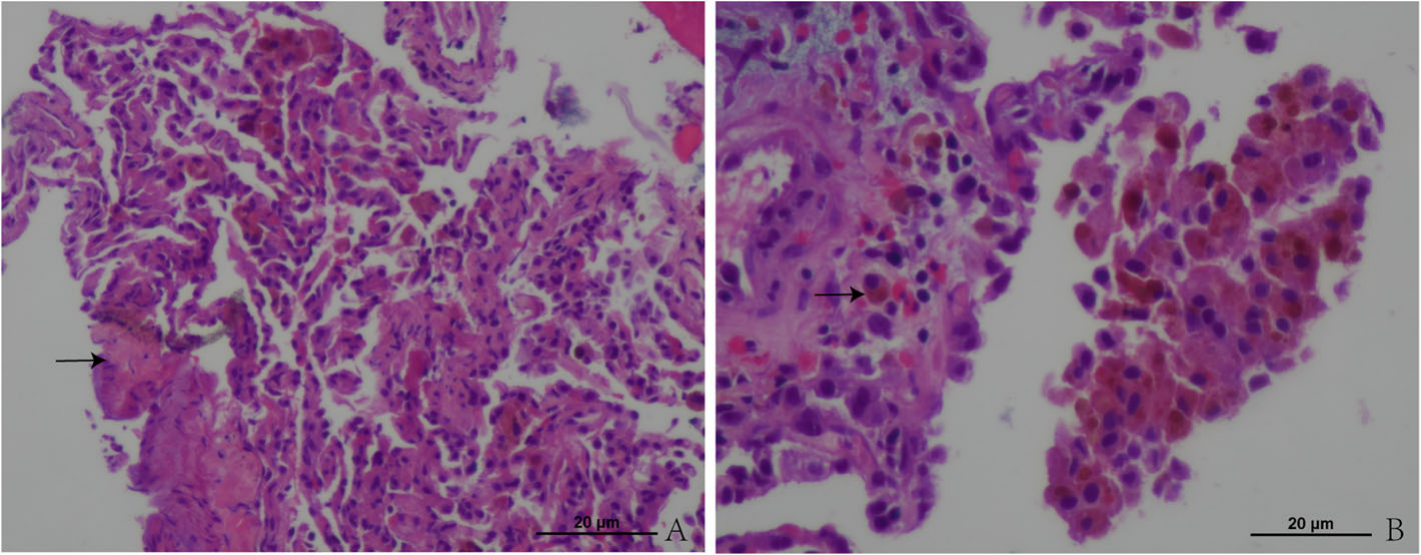
Histopathological investigation of the right lower lung nodules. **a** The alveolar tissue displayed mild atelectasis and hyalinization. HE staining: X100 magnification. **b** Haemosiderin-containing macrophages were observed. HE staining: X200 magnification

After 1 month, the pneumothorax relapsed, and the patient was readmitted to the hospital. Chest CT scans revealed bilateral pneumothorax with the mediastinum deviated to the right, and patch-like high-density shadows with an ambiguous boundary scattered in the two lungs (Fig. 1g, h and i). He was treated conventionally with an intercostal drain. Laboratory tests for connective tissue disease were performed again, and auto-antibodies were still negative. Bronchoscopy was performed again, and visible endobronchial lesions were not detected. An analysis of the bronchoalveolar lavage fluid (BALF) revealed a red blood cell count of 1270 cells/μL and a white blood cell count of 590 cells/μL (neutrophils 36%, lymphocytes 7%, monocytes 3% and other cell types 54%), prompting suspicion of occult intrapulmonary haemorrhage. Because of repeated relapses of pneumothorax and the presence of intrapulmonary nodules, the patient was referred for CT-guided lung puncture. A histopathological investigation of the right lower lung nodules revealed mild atelectasis and hyalinization of the alveolar tissue, and haemosiderin-containing macrophages were observed (Fig. 2). No specific indication of lung puncture pathology was found; he was discharged from the hospital and followed.

**Fig. 3:**
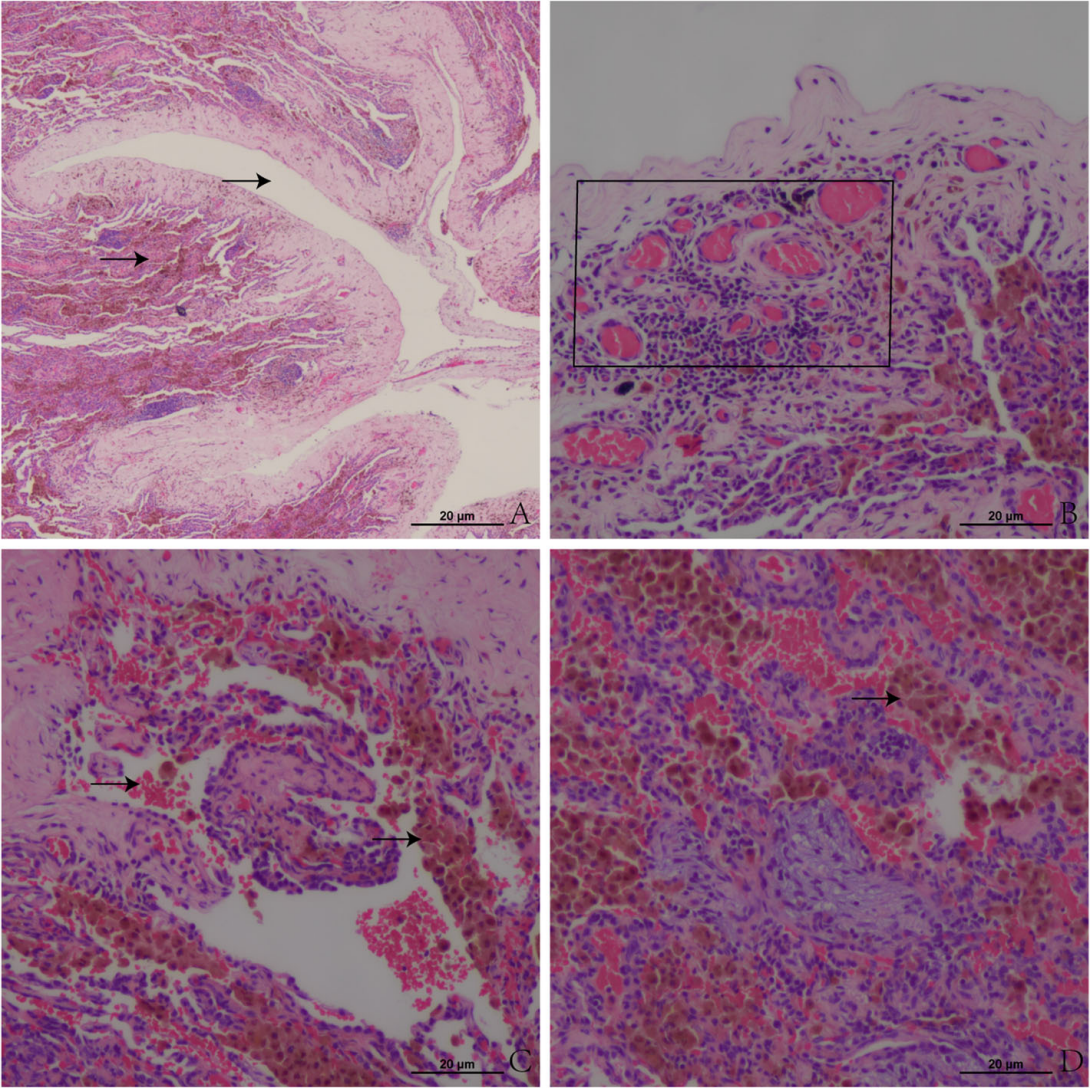
Pathological findings of the lung lesions resected through video-assisted thoracotomy. **a** The right arrow indicates the cavity, and the left arrow indicates the wall of the cavity containing granulation tissue. HE staining: X100 magnification. **b** The box indicates granulation tissue. HE staining: X200 magnification. **c** The arrows indicate fresh and old haemorrhages and intra-alveolar haemosiderin-containing macrophages, which indicated a previous haemorrhage. HE staining: X200 magnification. **d** Note the accumulation of haemosiderin-containing macrophages. HE staining: X400 magnification

In the next month, the pneumothorax relapsed again, and chest CT scans revealed gas on both sides of the chest cavity, scattered nodules in both lungs, and patchy high-density shadows with unclear boundaries (Fig. 1j, k and l). We performed wedge resection of the pulmonary parenchyma using video-assisted thoracoscopic surgery for his recurrent pneumothorax and obtained a specimen of the right lower lung lesion for pathology. The histopathological investigation revealed fresh and old haemorrhages and fibrous nodules. The wall of the cavity showed granulation tissue and calcification. Intra-alveolar haemosiderin-containing macrophages were indicative of a previous haemorrhage (Fig. 3).

**Fig. 4:**
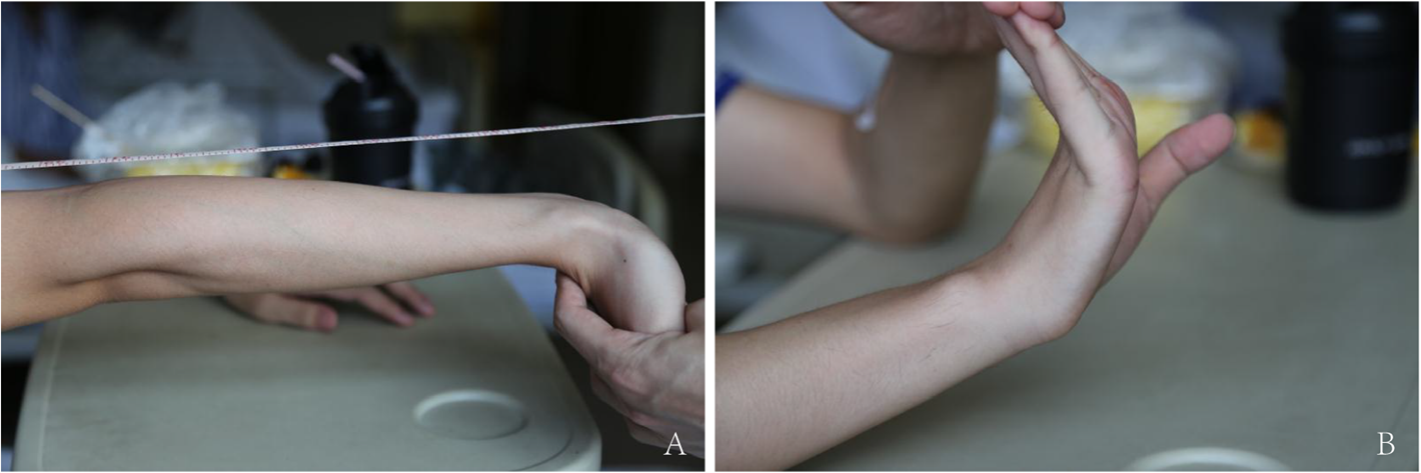
Hypermobility of the elbow and metacarpophalangeal joints. Hypermobility of the elbow greater than 10°

An in-depth physical examination was performed to determine the underlying cause of these atypical clinical manifestations. His skin was thin and translucent with visible venous patterns on the dorsal side of his feet. The clinical examination revealed subtle skin hyperextensibility on the face and forearm and hypermobility of the elbow and metacarpophalangeal joints (Fig. 4). No evidence of scoliosis and no history of joint dislocations were observed. These findings led us to suspect a diagnosis of vEDS.

Genomic deoxyribonucleic acid (DNA) was extracted from a blood sample collected from the patient. Whole-exome sequencing analysis was performed on the DNA sample using chip capture high-throughput sequencing at Shenzhen Huada Clinical Laboratory Center, Shenzhen, China. Chip capture high-throughput sequencing was performed to sequence exons and adjacent exon sequences of approximately 20,000 genes in the human genome. A variant analysis was performed on 3583 genes in the Online Mendelian Inheritance in Man (OMIM) database that had a clear correlation with a single genetic disease. The process of analysis involved first filtering the sequences by 1% of the population frequency (variants present in greater than 1% of the population frequency were evaluated as sites with a low probability of aetiology). According to the patient’s clinical complaint, we next assessed whether mutation sites were likely to fit the symptoms in the filtered mutation dataset. The pathogenicity of the variants was interpreted and classified according to the American College of Medical Genetics (ACMG) Guidelines published in 2015 [3].

Heterozygosity was observed for a novel variant, c.2977G > A (NM_000090.3), which represented a missense mutation that changes a glycine to serine at amino acid 993 (p. Gly993Ser) in the collagen type III alpha 1 protein. The mutation is named according to the Human Genome Variation Society (HGVS) recommendations [4]. This variation was not detected in gnomAD. The variant (COL3A1; NM_000090.3; c.2977G > A; p. Gly993Ser) was validated by Sanger sequencing in the proband and his parents. It was not detected in either of his parents, indicating that this variant was a de novo mutation. Other genes that caused overlapping features (such as FLCN) were excluded. Finally, a definitive diagnosis of vEDS was established.

Considering the deadly arterial complications of this disease, we then recommended further imaging tests to evaluate for possible arterial complications. The patient was referred for a magnetic resonance imaging (MRI) angiography of the intracerebral, thoracic and abdominal arteries, which showed normal calibres of all arteries.

However, 1 month after surgery, the patient experienced a left-sided pneumothorax that was treated with a chest tube again, and he was closely monitored in the clinic. Fortunately, as of 8 months after discharge, the patient remains symptom-free and lives well, without a relapse of pneumothorax or new pulmonary lesions.

## Discussion and conclusions

Primary spontaneous pneumothorax is a very common disease of the respiratory system that usually occurs in young and thin males. When pneumothorax develops, oxygen inhalation and thoracic puncture drainage often achieve satisfying results. However, the frequent occurrence of pneumothorax in a short time is rare. The list of differential diagnoses for pneumothorax is extensive (Table 1, data from reference [5]). When this young patient developed pneumothorax, unusual lesions in the lungs captured our attention. In fact, we considered the differential diagnoses for pneumothorax and suspected a variety of rare pulmonary diseases, including eosinophilic pneumonia, Langerhans cell histiocytosis, idiopathic pulmonary haemosiderosis, vasculitis or other interstitial lung diseases. Few eosinophils were detected in the patient’s blood and bronchoalveolar lavage fluid, and no eosinophils were observed in the lung tissue biopsy. Auto-antibodies were not detected in the blood. The histopathological investigation of the right lower lung biopsy specimen revealed fresh and old haemorrhage and did not show any evidence of vasculitis. The patient did not experience anaemia. A physical examination revealed that his skin was thin, transparent and hyperextensible, and the joints were hypermobile. All of these findings led us to suspect the diagnosis of vEDS. We waited 2 months for the results of the genetic report, and it revealed a mutation in the COL3A1 gene, thus confirming the diagnosis of vEDS. A detailed physical examination was very important, and the lengthy wait for the results of the genetic report was worthwhile.

**Table 1:** Aetiologies of pneumothorax

**Primary spontaneous pneumothorax**	
Associated with male sex, increased height, thin body habitus, and smoking	
**Secondary pneumothorax**	
Airway diseases	
Chronic obstructive pulmonary disease	
Cystic fibrosis	
Asthma	
Infectious causes	
Tuberculosis	
*Pneumocystis jirovecii* pneumonia	
Necrotizing bacterial pneumonia	
Interstitial lung disease	
Idiopathic pulmonary fibrosis	
Lymphangioleiomyomatosis	
Langerhans cell histiocytosis	
Lymphocytic interstitial pneumonia	
Sarcoidosis	
Connective tissue disease	
Ankylosing spondylitis	
Sjögren syndrome	
Rheumatoid arthritis	
Scleroderma	
Marfan syndrome	
Ehlers-Danlos syndrome	
Neoplasm	
Bronchogenic carcinoma	
Metastatic disease	
Sarcoma	
Miscellaneous	
Catamenial pneumothorax	
Birt-Hogg-Dubé syndrome	

vEDS results from structural defects or a deficiency in the pro-alpha 1 chain of type III procollagen encoded by the COL3A1 gene, which is a key component of many hollow organ tissues. Thus, this abnormal type III collagen synthesis is associated with hyperextensibility of the skin, joint hypermobility, and increased tissue fragility [6]. As shown in Table 2 (data from reference [7]), the international EDS consortium proposed a set of major and minor clinical criteria that are suggestive of vEDS diagnosis in 2017 [7]. A formal diagnosis of vEDS relies on molecular confirmation with the identification of a causative genetic variant. According to the 2017 diagnostic criteria for vEDS, this patient met three minor criteria: thin, translucent skin with increased venous visibility, spontaneous pneumothorax and hypermobility of small joints.

**Table 2:** Diagnostic criteria for vascular Ehlers-Danlos syndrome

Major criteria	
1. Family history of vascular Ehlers-Danlos syndrome with a documented causative variant in the COL3A1 gene	
2. Arterial rupture at a young age	
3. Spontaneous sigmoid colon perforation in the absence of known diverticular disease or other bowel pathology	
4. Uterine rupture during the third trimester in the absence of previous Caesarean section and/or severe peripartum perineum tears	
5. Carotid–cavernous sinus fistula (CCSF) formation in the absence of trauma	
Minor criteria	
1. Bruising unrelated to identified trauma and/or in unusual sites, such as the cheeks and back	
2. Thin, translucent skin with increased venous visibility	
3. Characteristic facial appearance	
4. Spontaneous pneumothorax	
5. Acrogeria	
6. Talipes equinovarus	
7. Congenital hip dislocation	
8. Hypermobility of small joints	
9. Tendon and muscle rupture	
10. Keratoconus	
11. Gingival recession and gingival fragility	
12. Early onset varicose veins (younger than age 30 and nulliparous if female)	

Minimal criteria suggestive for vEDS: A family history of the disorder, arterial rupture or dissection in individuals aged less than 40 years, unexplained sigmoid colon rupture, or spontaneous pneumothorax in the presence of other features consistent with vEDS should all lead to diagnostic studies to determine if the individual has vEDS. Testing for vEDS should also be considered in the presence of a combination of the other “minor” clinical features listed above

In our case, the patient exhibited a missense mutation c.2977G > A in the COL3A1 gene, which, to our knowledge, had never been reported. Missense mutations in the COL3A1 gene are more likely to be classified as pathogenic rather than benign in the ClinVar database, and the variant c.2977G > A detected in the proband was also a missense mutation. It changed a glycine to serine at amino acid 993 (p. Gly993Ser), which was predicted to be damaging by many computational algorithms, such as DANN, DEOGEN2, EIGEN, FATHMM-MKL, M-CAP, MVP, MutationAssessor, MutationTaster, PrimateAI, REVEL and SIFT. The results from the computational algorithms were obtained from a public interpretation platform named varsome [https://varsome.com/]. The variant is located in exon 41, the region encoding chains of the triple helical domain of type III procollagen [8], and two other missense variants in the same codon, Gly993Cys and Gly993Asp, have been reported to be pathogenic [9], indicating that this site is essential for the function of the COL3A1 gene. In addition, this variant was a de novo mutation in the proband and was consistent with his symptoms, suggesting that it provided a moderate support for the pathogenicity. Finally, we classified this variant as likely pathogenic, based on the evidence described above.

Mutations that cause a glycine substitution in the triple helical protein domain might damage the structural integrity of collagen molecules [10]. As a result, the production of mature type III collagen is substantially reduced and subsequently reduced the mechanical strength of arteries and other hollow organs [11, 12].

Up to 80% of patients with vEDS may have a life-threatening vascular or viscus rupture before the age of 40 years [1]. Although lethal complications had not yet occurred in this patient, the risk of developing dissection, aneurysms, and rupture of arteries later in life was significant. As reported in the study by Kumagaya et al., a patient diagnosed with vEDS initially presented with recurrent pneumothorax and intrapulmonary lesions without vascular complications; however, an aneurysmal formation of the left ulnar artery developed 7 years later, and an aneurysmal formation or arterial dilation of the right ulnar artery, celiac artery, and left iliac artery were observed 12 years later [13]. Our patient was informed about the risk of bleeding in the lungs and other organs later in life.

This patient was treated with the placement of a chest tube and wedge resection of the lungs before a clear diagnosis was established. However, the placement of chest tube, pleurodesis or wedge resection may be complicated by lethal haemothorax in patients with vEDS, and thus a conservative approach should always be used if at all possible.

Celiprolol is a unique β-blocker with β1-adrenergic receptor antagonism and partial β2-agonist activity that results in a lower pulse pressure and heart rate. As shown in the study by Frank et al., untreated patients displayed a significantly worse outcome than patients treated with celiprolol (survival rate of 72.4% vs. 52.2%; *p* < 0.001) [14]. In terms of the drug dosage, 400 mg/day should be considered the optimal treatment dose [14].

Patients with vEDS rarely present with multiple pneumothoraces. In this case, we observed recurrent pneumothoraces, pulmonary consolidation and cavities, which probably resulted from the rupture of blebs and lung lesions. The lungs of patients with vEDS are vulnerable to laceration.

The reasons why we present this case are listed below. 1. vEDS is a rare disease and patients rarely present with multiple pneumothoraces. 2. We report a patient with a novel missense mutation in the COL3A1 gene (NM_000090.3: c.2977G > A).

When a patient initially presents with pulmonary complications, vEDS is usually not suspected. However, when a patient presents with recurrent pneumothorax, intrapulmonary cavities and nodular lesions, and a physical examination reveals thin and transparent skin, hypermobility of joints, vEDS should be considered. Considering the severity and rapid progression of vEDS, a molecular diagnosis is crucial.

## Data Availability

All the data regarding the findings are available within the manuscript.

## Abbreviations

vEDS: Vascular Ehlers-Danlos syndrome
CT: Computed tomography
CTA: Computed tomography angiography
HRCT: High-resolution computed tomography
BALF: Bronchoalveolar lavage fluid
DNA: Deoxyribonucleic acid
OMIM: Online Mendelian Inheritance in Man
ACMG: American College of Medical Genetics
HGVS: Human Genome Variation Society
MRI: Magnetic resonance imaging
